# Changes in nail position and antirotation blade angles on the risk of femoral head varus in PFNA fixed patients: a clinical review and comprehensive biomechanical research

**DOI:** 10.1186/s40001-024-01892-7

**Published:** 2024-06-18

**Authors:** Chenyi Huang, Wenqiang Xu, Xiong Ye, Wanying Hong, Yue Xu, Zongchao Liu, Jingchi Li

**Affiliations:** 1 Department of Orthopedics, Luzhou Key Laboratory of Orthopedic Disorders, The Affiliated Traditional Chinese Medicine Hospital, Southwest Medical University, No. 182, Chunhui Road, Luzhou, 646000 Sichuan Province People’s Republic of China; 2 Department of Orthopaedics, Affiliated Hospital of Integrated Traditional Chinese and Western Medicine, Nanjing University of Chinese Medicine, Nanjing, 210028 Jiangsu Province People’s Republic of China; 3 Department of Orthopaedics, Changshu Hospital of Traditional Chinese Medicine, Changshu, 215500 Jiangsu Province People’s Republic of China

**Keywords:** Intertrochanteric fracture, Proximal femoral nail anti-rotation, Fixation stability, Intramedullary nail position changes, Femoral head varus

## Abstract

**Background:**

Femoral head varus triggers poor clinical prognosis in intertrochanteric fracture patients with proximal femoral nail antirotation (PFNA) fixation. Studies present that changes in nail position and screw insertion angles will affect fixation stability, but the biomechanical significance of these factors on the risk of femoral head varus has yet to be identified in PFNA fixed patients.

**Methods:**

Clinical data in PFNA fixed intertrochanteric fracture patients have been reviewed, the relative position of intermedullary nail has been judged in the instant postoperative lateral radiography. Regression analyses have been performed to identify the effect of this factor on femoral head varus. Corresponding biomechanical mechanism has been identified by numerical mechanical simulations.

**Results:**

A clinical review revealed that ventral side nail insertion can trigger higher risk of femoral head varus, corresponding numerical mechanical simulations also recorded poor fixation stability in models with ventral side nail insertion, and changes in the trajectory of anti-rotation blade will not obviously affect this tendency.

**Conclusions:**

Ventral side insertion of intramedullary nail can trigger higher risk of femoral head varus in PFNA fixed patients by deteriorating the instant postoperative biomechanical environment, and changes in blade trajectory cannot change this tendency biomechanically. Therefore, this nail position should be adjusted to optimize patients’ prognosis.

## Background

Intertrochanteric fracture is a common type of osteoporotic fracture in elder patients [1, 2]. With the aggravation of aging population tendency, the fracture incidence stepwise increased. Internal fixation operation is an effective method to treat the intertrochanteric fracture, and several different fixation devices have been used in the fracture treatment in which the proximal femoral nail antirotation (PFNA) is the most widely used fixation device and achieved definite clinical effect [3, 4]. However, as a hardware related complication, femoral head varus can trigger patient’s poor prognosis. The essence of this complication is the cutting of bony structure by anti-rotation blade under weight-bearing conditions, or in other words, is a kind of fixation instability [5, 6]. Therefore, any potential strategy which can promote the fixation stability may be an alternative method to reduce the risk of femoral head varus.

Biomechanical significance of fixation device position and angle changes on fixation stability have been widely reported. In intramedullary fixation, changes in nail insertion position will affect the fixation stability. Changes in nail insertion position (ventral or dorsal sides movement) are common in PFNA operation, but its biomechanical significance on fixation stability and femoral head varus risk has yet to be identified [4, 7]. Meanwhile, cross fixation is also an effective method to optimize fixation stability. However, there is also no relevant report on whether the cross fixation of anti-rotation blade should be performed in PFNA fixed patients with forward or backward changes in nail insertion [8, 9]. In this study, by performing a comprehensive research consisted by clinical review, mechanical tests and numerical simulations [10, 11], the significance of nail insertion position movement and blade cross fixation on fixation stability and potential risk of femoral head varus have been identified. To our knowledge, this was the first study to identify this topic.

## Materials and methods

### Clinical review

#### Patient collection

The ethics committees of our hospital reviewed and approved the protocol of this study. Informed consent was waived for this retrospective study. We retrospectively reviewed patients who suffered from the intertrochanteric fractures and underwent PFNA fixation from August 2020 to September 2021. The exclusion criteria were as follows: (1) had femur trauma or an operation history; (2) had a pathological fracture caused by primary or metastatic bone tumors, bone tuberculosis, or rheumatic immune diseases; (3) underwent revision surgery within the clinical follow-up period of 6 months for other complications; (4) had conservative treatment; (5) were lost to follow-up or died during the follow-up period; and (6) had long-term bed rest. Patient demographic parameters, including age, sex, bone mineral density (BMD) (T-score measured by DXA) and body mass index (BMI), were recorded. A well-trained orthopedic surgeon performed all the PFNA operations, and the tip-to-apex distance (TAD) for the anti-rotation blade was < 25 mm for all the enrolled patients. Patient’s demographic data, including their age, sex and body mass index, have been recorded according to their medical record [12, 13].

### Radiographic data collection

**Fig. 1:**
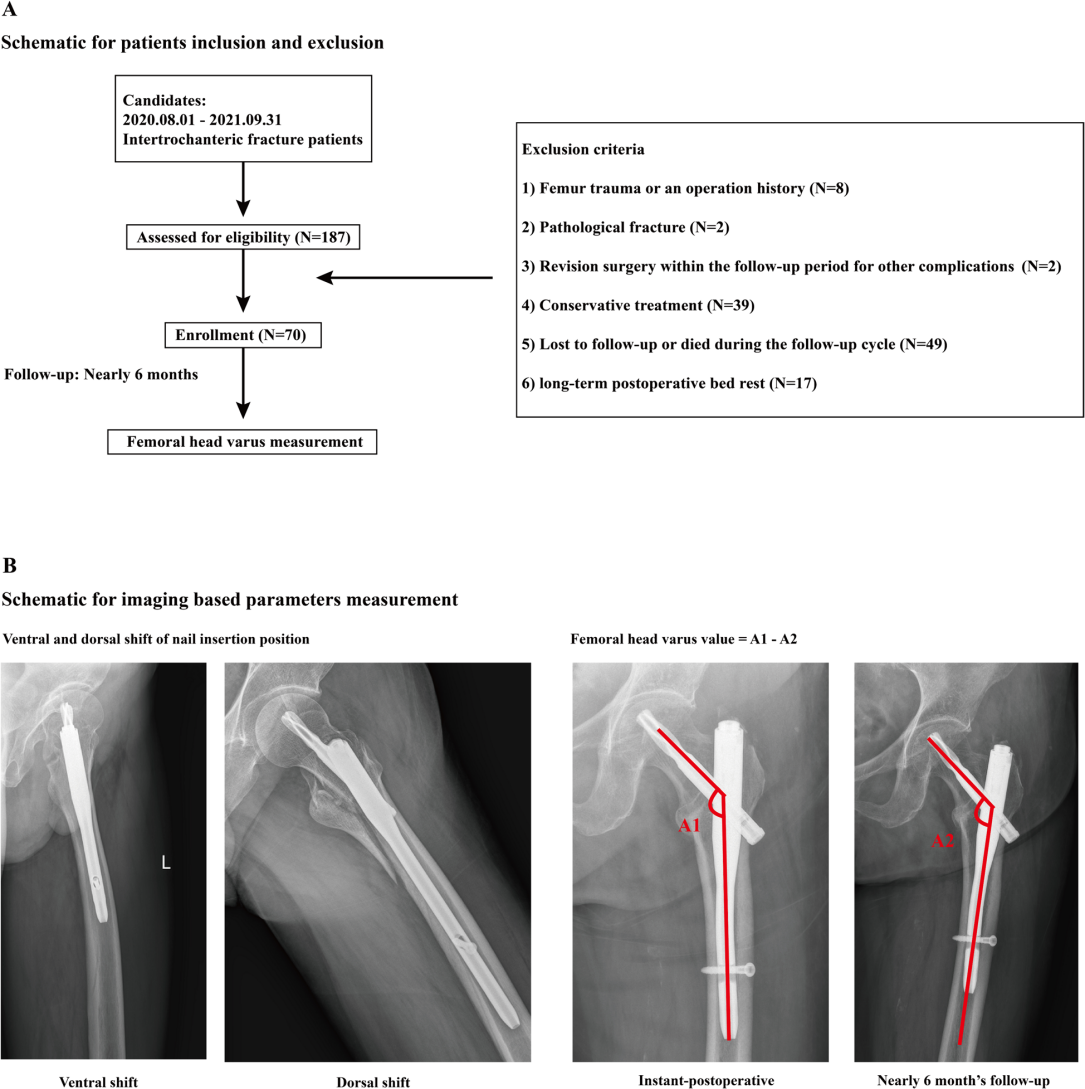
Schematic of patient inclusion and exclusion and measurement of imaging-based parameters

All patients underwent anterior–posterior radiography three times, including immediately before and after the operation and nearly 6 months after the screw fixation operation. Imaging data measurement have been performed by an experienced orthopaedic surgeon. As an early stage complication, the currently selected follow-up period can well represent the grade of femoral head varus progression [9, 14]. The femoral neck–trunk angle was measured via anterior–posterior radiography at two different times, and the difference between these angles was computed to represent the varus status of the femoral head [9, 14]. The position of intramedullary nail was judged in the instant-postoperative lateral radiography. In addition, the nail position was divided into two groups: ventral and dorsal sides (Fig. 1) [15, 16].

### Statistical analyses

We conducted the statistical analyses with SPSS software. Radiographic and demographic indicators are presented as the mean ± standard deviation for continuous variables and as the number (percentage) for categorical variables [17, 18]. One week after the measurement of imaging parameters, 20 patients were randomly selected. Parameters for these patients were re-measured by the orthopaedic surgeon and another well-trained surgeon. The intraclass correlation efficiency (ICC) was computed to determine the repeatability of femoral head varus values [19–21], and the Kappa value was computed to determine the repeatability of nail position judgement. ICC and Kappa values ≥ 0.8 represent excellent reliability [22, 23]. We performed linear regression analysis to identify independent risk factors for femoral head varus [24, 25]. Univariate analyses of each potential risk factor were performed, and the variables that achieved a significance level of *p* < 0.1 were entered into multivariate analyses. Variables with *P* < 0.05 were considered independent risk factors in the multivariate analysis [26, 27].

## Numerical mechanical simulations

### Construction of the intact finite-element (FE) model

The proximal femur model was constructed based on the outline of the syn-bone model rather than that of any special patient. The model construction strategy was selected to avoid ethics-related procedures and eliminate the confounding effects caused by individual differences in the outlines of different patients [14, 28]. A thin CT scan was performed on the syn-bone femur model (thickness = 0.55 mm). The range of the proximal femur was defined as the distance from the tip of the femoral head to 30 cm below the lesser trochanter [14, 29]. The outline of the proximal femur model was constructed according to the CT-scanned femur outline in 3D-CAD software. This study consisted of our published studies. The computational efficiency and accuracy of the numerical model constructed by this method were better than those of the traditional reverse model construction strategy [30, 31]. Cortical and cancellous bones were separately constructed, and outlines of these bony structures were constructed separately based on the CT imaging data.

### Construction of PFNA fixed intertrochanteric fracture models with different nail positions and blade trajectories

To simulate the fixation stability of different screw fixation strategies in the instability fracture model, the AO 3-1 2.3 type intertrochanteric fracture model was constructed [29, 32]. The numerical model of a PFNA fixation device was also constructed with 3D-CAD software. When simulating the PFNA fixation operation, in the control group, the intramedullary nail was inserted along the central point of femoral shaft on the transverse plane, the anti-rotation blade was parallel to the axis of femoral neck in both coronal and sagittal plane [33, 34].

**Fig. 2:**
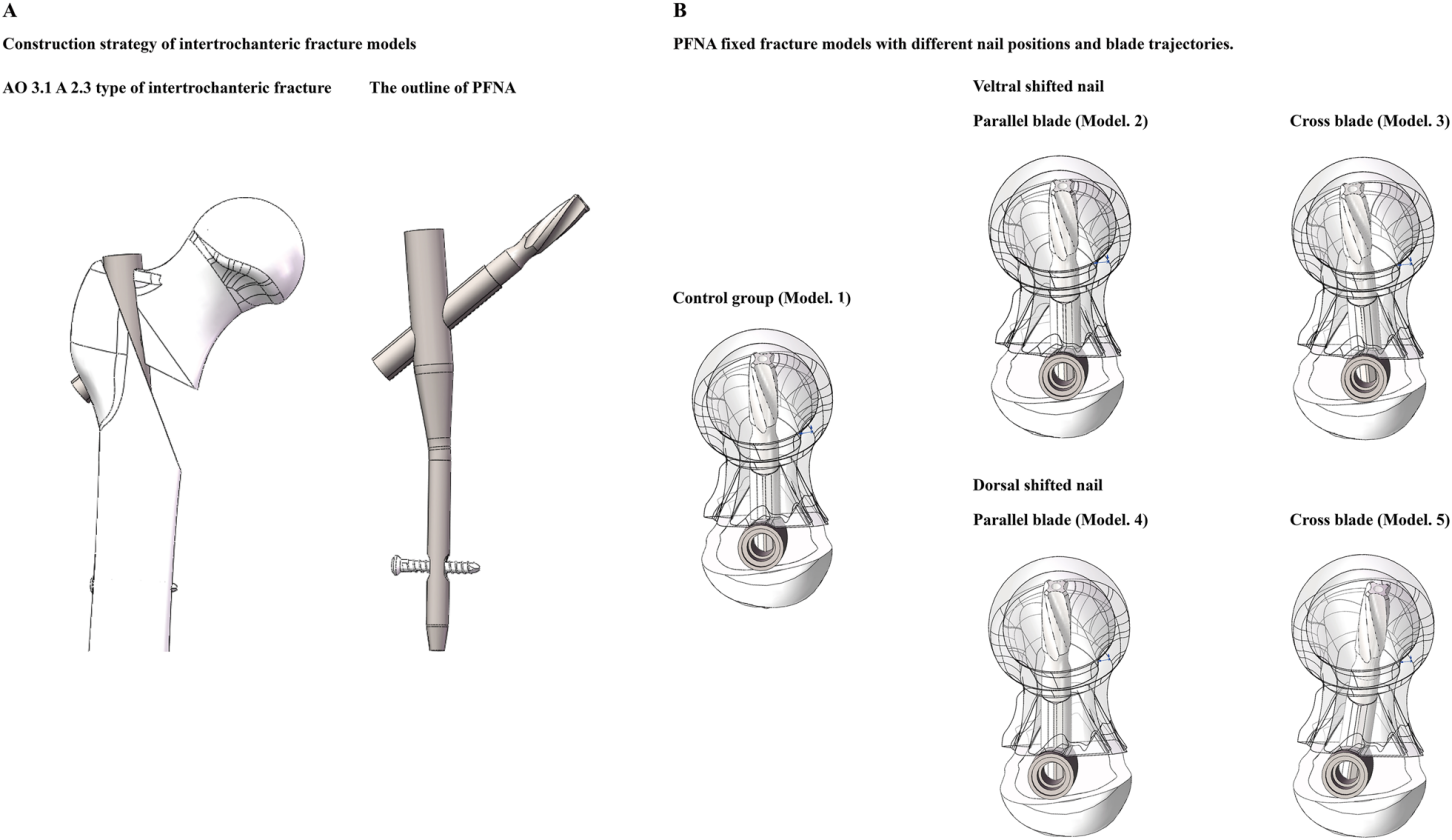
Schematic for the construction of PFNA fixed AO 3.1 A 2.3 type of intertrochanteric fracture models with different nail positions and anti-rotation blade trajectories

To investigate the biomechancial significance of nail position changes, the insertion point of intramedullary nail was shift 3 mm to both ventral and dorsal sides from that of the control group. Moreover, to investigate if the biomechanical significance of nail position changes can be affected by changes in anti-rotation blade changes, two different blade trajectories (parallel, and cross trajectories) were simulated in models with both ventral and dorsal sides nail insertion. In models with parallel blade trajectory, the axis of blade was parallel to that of the femoral neck. In contrast, in models with cross blade trajectory, the axis of the blade was defined to the opposite of the femoral head (i.e., in the model with ventral position nail, the axis of blade was oriented to the dorsal side of femoral head, and vice versa). Using this model construction strategy, five different numerical models (i.e., control group, ventral and dorsal nail positions with parallel and cross blade trajectories) have been constructed (Fig. 2).

### Boundary and loading conditions

Different sizes of tetrahedron element were selected in different components of postoperative models. Smaller mesh sizes were selected on bone-blade interfaces to reduce the incidence of mesh deterioration. Using this mesh generation strategy, the average mesh quality was > 0.8. According to the same type studies, this mesh quality can ensure the computational credibility [10, 13]. The material properties of cortical and cancellous bone and the titanium alloy cannulated screw were separately defined as isotropic material according to the same type of study. The degrees of freedom of the inferior surfaces of the numerical models were completely fixed. Compressive load were applied to the femoral head at 10° lateral to the coronal plane and 9° posteriorly to the sagittal plane [14, 29]. A 2100 N load was applied to compute the deformation and stress of the femoral head during daily activity for patients with 70 kg’s body weight [35, 36]. Stress distribution on the anti-rotation blade and femoral head, and the maximum deformation of femoral head have been computed under this loading condition. Moreover, the compressive load value was recorded when the maximum deformation of the femoral head reached 10 mm, and the corresponding force was recorded as the failure strength of the screw fixation [35, 36].

## Results

### Clinical review

**Fig. 3:**
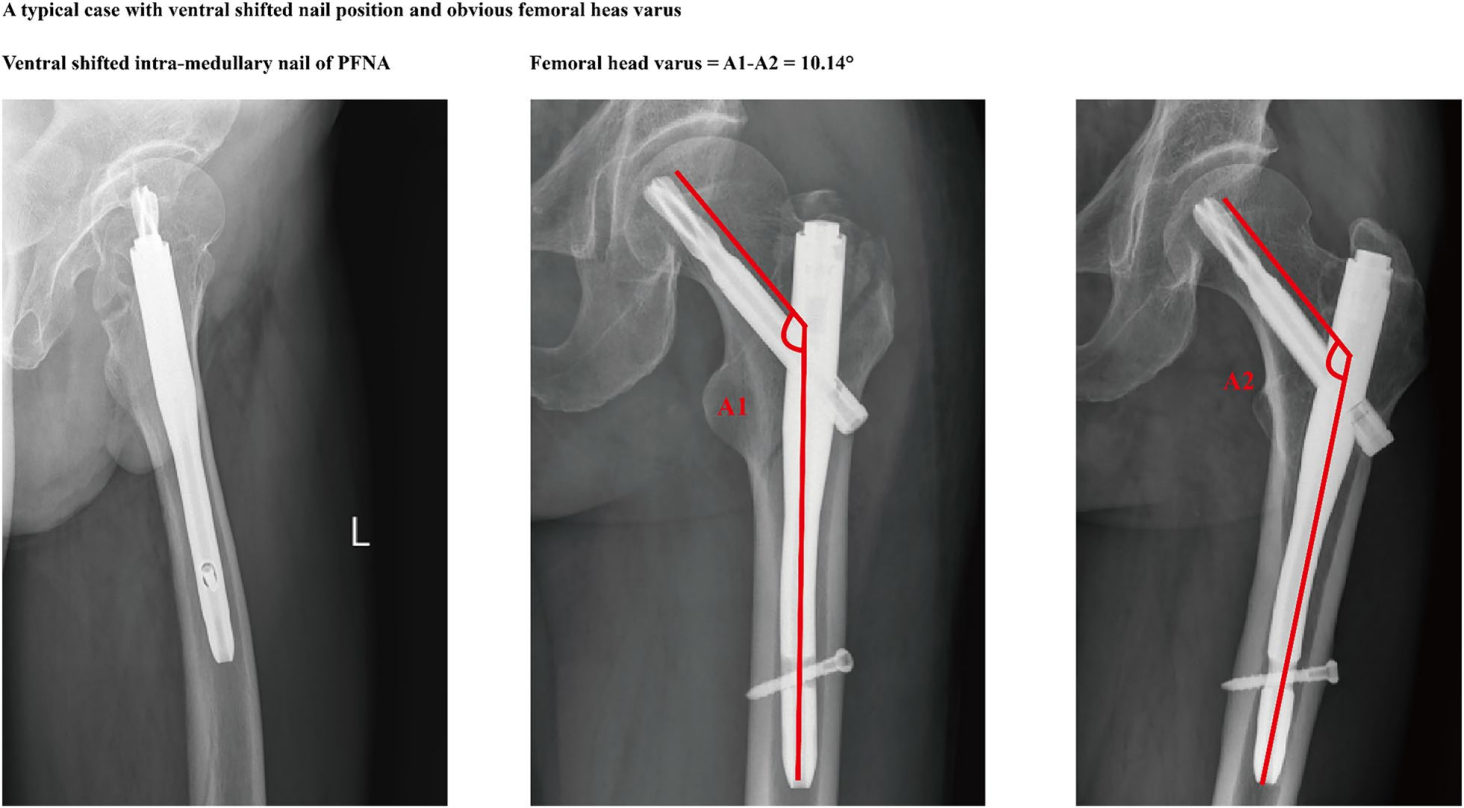
Typical case with ventral shifted intramedullary nail position, and obvious femoral head varus during the 6 month follow-up period

Clinical data of 70 patients were enrolled in this study. Excellent inter- and intraobserver reliability can be observed when imaging-based parameters are measured. Inter- and intraobserver ICC values when measuring the femoral head varus were 0.912 and 0.887, respectively (Table 1). Besides, ideal reliability can be recorded when judging the relative position of intramedullary nail. Inter- and intraobserver Kappa values were 0.8 and 0.9 in this process. According to results of the univariate regression analysis, *P* values of the patient’s demographic parameters, including the age, sex BMI were > 0.1, and that of BMD and nail position changes was < 0.1. Therefore, these factors were enrolled into multivariate regression analysis, and only the ventral shifted nail position was proved to be an independent risk factor for femoral had varus. In contrast, a critical positive result can be recorded for the reduction of BMD (*P* = 0.076) (Table 2 and Fig. 3).

**Table 1:** ICC and Kappa values of inter- and intraobserver reliability when measuring imaging based parameters

	Interobserver	Intraobserver
Femoral head varus values	0.912	0.887
Intra-medullary nail positions	0.8	0.9

**Table 2:** Linear regression analysis of severe femoral head varus

	t	95% CI		*P* value
*Uni-variable analyses*				
Age	− 1.264	− 0.036	0.008	0.213
Sex (Male: 1, Female: 2)	− 0.954	− 0.860	0.907	0.345
BMI	1.568	− 0.029	0.234	0.124
BMD	− 2.828	− 0.753	− 0.126	0.007^#^
Intramedullary nail position (1. Dorsal shift; 2. Ventral shift)	3.182	0.299	1.335	0.003^#^
*Multi-variable analyses*				
BMD	− 1.818	− 0.619	0.032	0.076
Intramedullary nail position (1. Dorsal shift; 2. Ventral shift)	2.28	0.071	1.17	0.028*

^#^Variables that achieved a significance level of *p* < 0.1 in the univariate analysis

^*^Statistical significance (*P* < 0.05)

^**^Statistical significance (*P* < 0.01)

### Numerical mechanical simulations

**Fig. 4:**
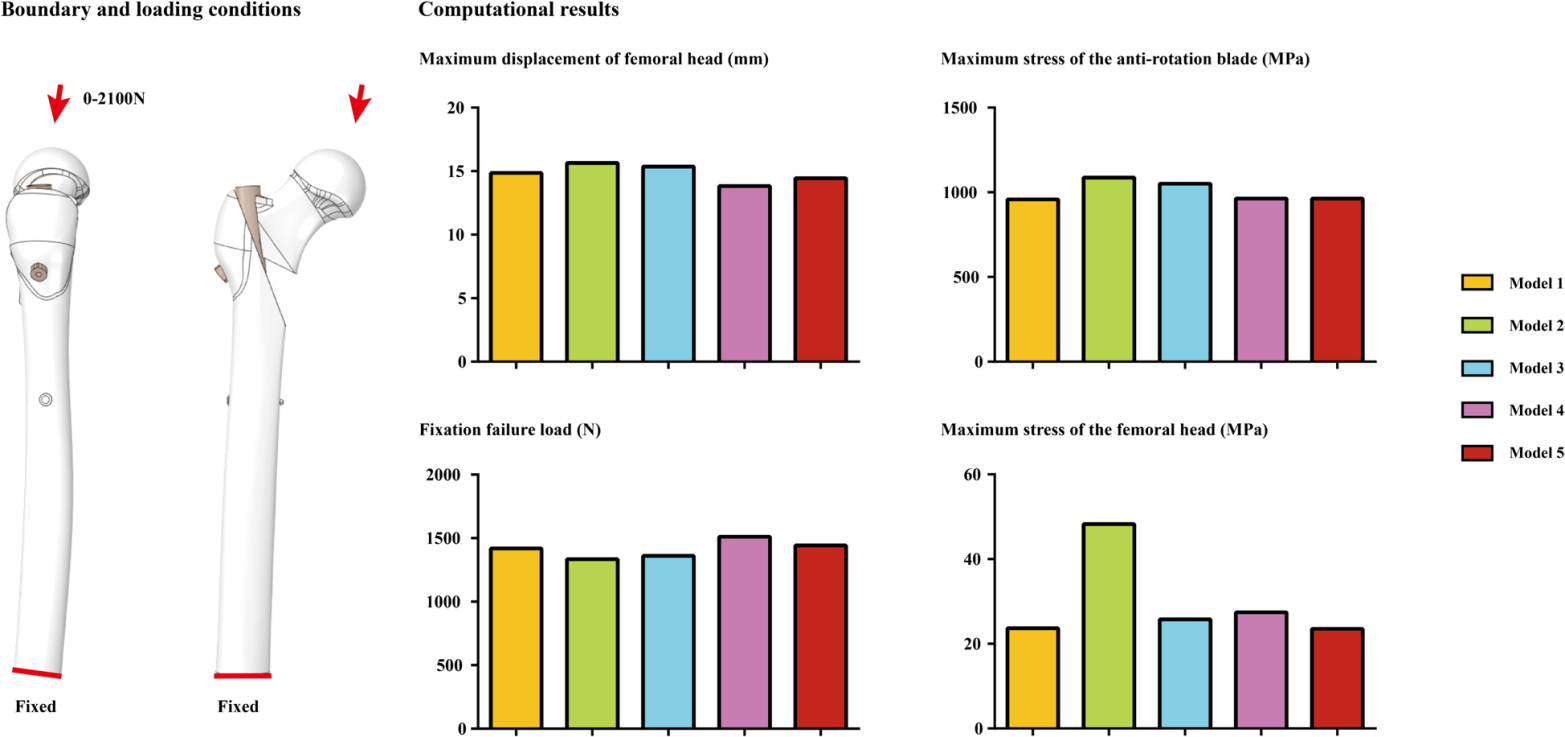
Boundary and loading conditions of numerical simulations and computational results obtained using different models

**Fig. 5:**
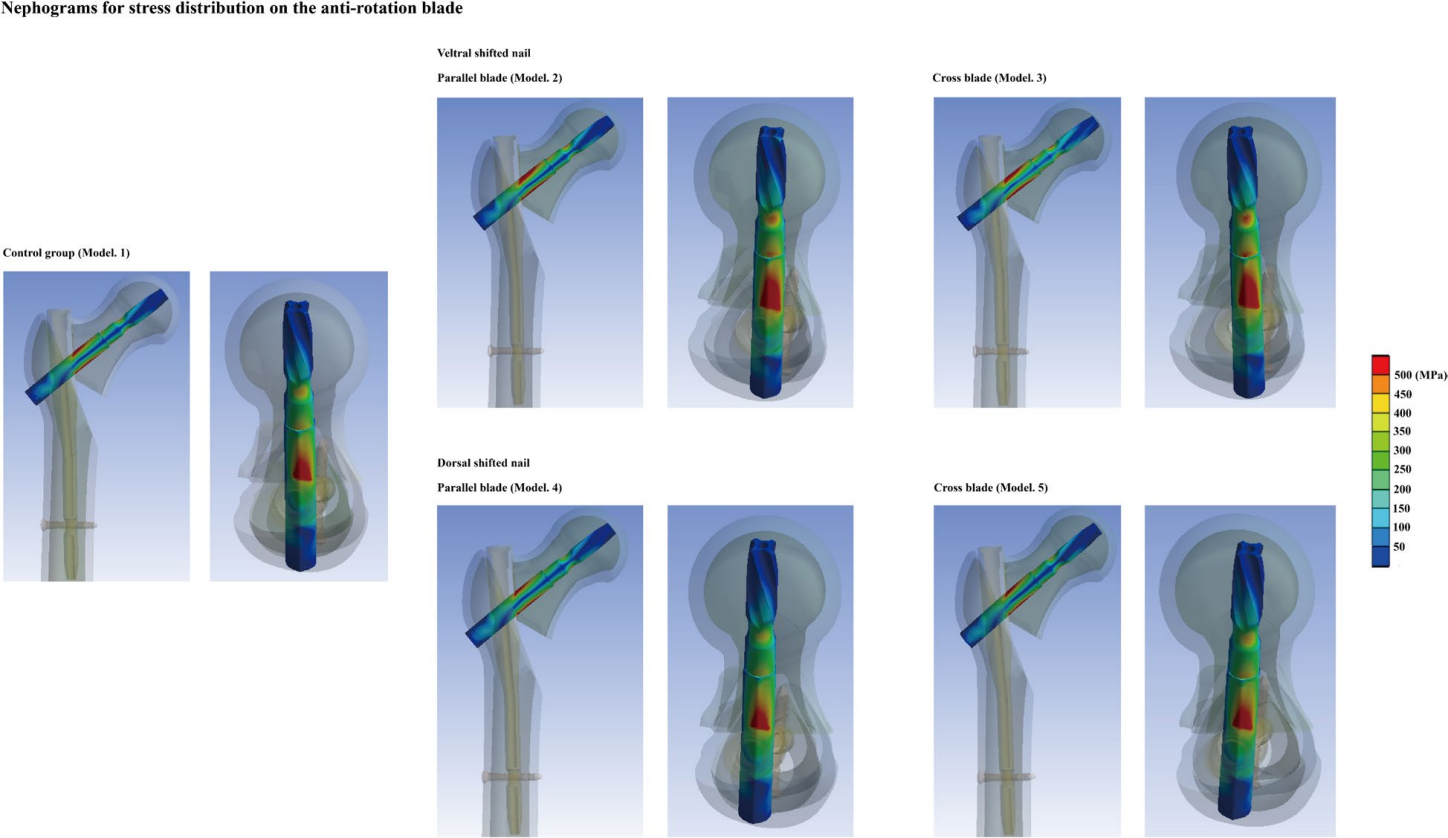
Nephograms of stress distributions on the anti-rotation blade under 2100N compressive load

To investigate the biomechanical significance of nail insertion position changes, the maximum deformation of femoral head, the maximum stress of anti-rotation blade, and femoral head under the 2100N compressive load, ant the failure fixation load have been computed and recorded in this study. A overall consistent variation tendency can be observed in different models. Specifically, compared to the control group model and models with dorsal shift of nail insertion position, higher stress value and poor fixation stability can be observed in models with ventral shift of nail insertion position. The maximum stress value of the model with ventral shifted nail insertion position and parallel blade trajectory was 48.25 MPa, and that of all other models were < 30 MPa. Besides, the maximum stress of the anti-rotation blade in models with ventral shifted nail position was > 1000 MPa, and that in all other models was less than this stress value. Similarly, the maximum femoral head deformation under the 2100N compressive load was > 15 mm in models with ventral shifted nail, but less than this deformation value in other models. Finally, the failure load of models with ventral shifted nail was < 1400N, but larger than this load value in other models (failure load of the model with dorsal shifted nail and parallel blade trajectory even higher than 1500N). Moreover, cross blade trajectory indeed optimize the fixation stability, this procedure obviously reduce the maximum stress on the femoral head, and slightly reduce stress on the blade, and deformation on the femoral head, and slightly increase the failure load of PFNA fixation (Figs. 4 and 5).

## Discussion

Femoral head varus is a commonly observed phenomenon in patients with PFNA fixation. Higher femoral head varus can trigger the deterioration of hip joint function [4, 7]. Therefore, identifying potential risk factor for femoral head varus and reduce the corresponding risk by optimizing the intraoperative of PFNA is of significance to optimize patients prognosis. Besides, changes in biomechanical environment can directly affect the instant postoperative stability, biomechanical deterioration can trigger fixation instability and higher risk of postoperative complications in patients with internal fixation operation [35, 37]. Therefore, identifying the biomechanical mechanism caused by intraoperative procedure changes can directly guide the surgical optimization [38, 39].

Changes in nail insertion position is common in the intramedullary nail fixation operation, but whether this change can affect the instant postoperative biomechanical environment and corresponding risk of femoral head varus in patients with PFNA fixation has yet to be identified. In this study, to provide theoretical guidance for the optimization of PFNA fixation, a comprehensive research consisted by clinical review and numerical simulations have been performed. A consisted variation tendency can be observed in clinical review and biomechanical simulations: poor fixation stability and higher femoral head varus values can be observed in patients and models with ventral shifted nail. Therefore, when inserting the intra-medullary nail, the ventral side nail position should be avoided, or adjusted in the PFNA operation to reduce the risk of femoral head varus biomechanically.

Besides, changes in screw trajectory and screw insertion angles can also affect the fixation stability [40, 41], but the clinical and biomechancial significance of anti-rotation blade insertion angle changes on the risk of femoral head varus also yet to be identified. To investigate this topic, numerical models with parallel and cross blade trajectories have been constructed. Biomechanical computational results show that in models with poor fixation stability (i.e., in models with ventral shifted nail), cross blade trajectory can effectively reduce the stress concentration tendency on the bone-screw interface and optimize the fixation stability. Therefore, consisted to published studies, cross blade trajectory was also an alternative method to optimize the fixation stability, and reduce the risk of femoral head varus in PFNA fixed patients.

Moreover, although studies present that BMD reduction can trigger higher incidence of fixation failure [17, 18], this factor was not proved to be an independent risk factor for femoral head varus in the current study. The critical positive result in the linear regression analysis may root in the limited sample size. Moreover, the predictive performance of T-score based BMD measurement was limited, and which has also been reported by several studies. We will try some new BMD judgement parameters (e.g., HU value) to optimize the predictive performance when judging the risk of femoral head varus in our future studies [17, 18]. In addition, we still believe that the result of this study do not deny the significance of anti-osteoporosis therapy on the optimization of fixation stability [42, 43].

Following topics should be clarified from the methodological perspective. First, limited by the numerical model simulation method, the compaction of bony structure during the cyclic load process cannot be effectively simulated in current models [44, 45]. Therefore, only instant postoperative biomechanical environment (in the first time compressive load application) can be reflected in numerical models [46, 47]. To optimize the credibility of femoral head varus risk prediction, four different parameters, including the maximum stress of anti-rotation blade and femoral head, the maximum displacement value of femoral head, and the failure load of different model have been computed in this study. In which, stress values were computed to deduce the micro-fracture of bone structures around the screw trajectory during the cyclic load. In other word, stress distribution on the blade and femoral head can reflect potential risk of bony compaction and resulting fixation failure. Moreover, the maximum displacement of femoral head, and the fixation failure load can reflect the instant postoperative fixation stability. Therefore, by comprehensively computing these parameters, potential risk of femoral head varus can be effectively judged [48, 49].

Moreover, the outline of currently used femoral model was root in the standard saw-bone model, rather than any volunteer specific imaging data, although the latter one was the most commonly used model construction strategy, individual differences in morphological parameters negatively affect the computational credibility and repeatability [14, 29]. In contrast, using a standard model with identical outline, this limitation can be effectively overcome, and the radiation damage and ethical concerns caused by CT scans of healthy volunteers can also be eliminated. Therefore, increase number of same type studies select the standard model to construct the outline of numerical models [30, 31].

Admittedly, several limitations existed in the current study. Firstly, nail position judgement was performed based on the instant postoperative lateral radiography. Therefore, only nail position change can be judged on the sagittal plane. However, in the real intraoperative procedure, changes of nail position on the transverse plane may also affect the fixation stability. However, for the lack of instant postoperative three-dimensional CT imaging data, the clinical significance of this topic cannot be identified in the current study. Moreover, only one fracture type was simulated in current numerical models. When evaluating the biomechanical significance of surgical related parameters, researchers prone to construct instability fracture models. Using this method, the biomechanical effect of the research topic (the trajectory of anti-rotation blade in this study) can be effectively presented. In contrast, when evaluating the biomechanical significance of the surgical related parameter in model with stable types of fracture, the stability of fracture fragments itself may lead to false negative results. Therefore, we select the AO 3-1 2.3 type of fracture (unstable intertrochanteric fracture) to construct surgical models. And this model construction strategy was consisted to same type studies [9, 14].

In conclusion, by performing a comprehensive study consisted by clinical review and numerical mechanical simulations, we present that the ventral side insertion of intramedullary nail can trigger higher risk of femoral head varus in PFNA fixed patients by deteriorating the instant postoperative biomechanical environment, and changes in blade trajectory cannot change this tendency biomechanically. Therefore, this nail position should be adjusted to optimize patients’ prognosis. However, the credibility of this study still limited by above-mentioned defects. And the current research conclusion still should be re-validated in our future studies with larger sample sizes, complete imaging data, and numerical models with more fracture types.

## Conclusion

The ventral side insertion of intramedullary nail can trigger higher risk of femoral head varus in PFNA fixed patients by deteriorating the instant postoperative biomechanical environment, and changes in blade trajectory cannot change this tendency biomechanically.

## Data Availability

All the data of the manuscript are presented in the paper.

## Abbreviations

BMD: Bone mineral density
BMI: Body mass index
FE: Finite Element
ICC: Intraclass correlation efficiency
PFNA: Proximal femoral nail antirotation
